# Main Factors Influencing the Gut Microbiota of Datong Yaks in Mixed Group

**DOI:** 10.3390/ani12141777

**Published:** 2022-07-11

**Authors:** Wen Qin, Shuang Li, Nan Wu, Zhouxuan Wen, Jiuxiang Xie, Hongyi Ma, Shoudong Zhang

**Affiliations:** 1 State Key Laboratory of Plateau Ecology and Agriculture, Qinghai University, Xining 810016, China; drlizzzy@163.com; 2 Northwest Institute of Plateau Biology, Chinese Academy of Sciences, Xining 810001, China; lishuang@nwipb.cas.cn; 3 College of Ecological and Environmental Engineering, Qinghai University, Xining 810016, China; 200710040123@qhu.edu.cn (N.W.); wenzhouxuan@outlook.com (Z.W.); 4 College of Agriculture and Animal Husbandry, Qinghai University, Xining 810016, China; xiejiuxiang8817@163.com; 5 Forestry and Grassland Comprehensive Service Center of Yushu Prefecture, Yushu 815000, China; mhy13195771222@163.com; 6 Ministry of Education Key Laboratory for Biodiversity Science and Ecological Engineering, Coastal Ecosystems Research Station of the Yangtze River Estuary, School of Life Sciences, Fudan University, Shanghai 200433, China; 7 Global Flyway Ecology, Conservation Ecology Group, Groningen Institute for Evolutionary Life Sciences (GELIFES), University of Groningen, 9700 CC Groningen, The Netherlands

**Keywords:** Datong yak, *Bos grunniens*, gut microbiota, mix group

## Abstract

**Simple Summary:**

This study examined the differences and similarities in gut microbial diversity and ecological assembly processes of Datong yaks, including domestic males and females and wild males, which were fed together on the Qinghai-Tibet Plateau in a mixed group. The results revealed that mixed grouping could influence the gut microbiota of these three groups of yaks and improve the gut microbial diversity of domestic females. The findings of this study can help to understand the effects of mixed grouping on the gut microbiota of livestock on the Qinghai-Tibet Plateau and improve the production of Datong yaks.

**Abstract:**

The Datong yak (*Bos grunniens*) is the first artificial breed of yaks in the world and has played an important role in the improvement of domestic yak quality on the Qinghai-Tibet Plateau. The Datong yak breeding farm in the Qinghai province of China is the main place for the breeding and feeding of Datong yaks. It hosts domestic Datong yaks and wild male yaks, mainly in mixed groups. Different managements have different effects on livestock. The gut microbiota is closely related to the health and immunity of Datong yaks, and mixed grouping can affect the composition and diversity of the gut microbiota of Datong yaks. To reveal the effects of mixed grouping on the gut microbiota of Datong yaks and wild yaks and identify the main dominant factors, we compared the gut microbial diversities of domestic males and females and wild males based on 16S rRNA V3–V4 regions using fresh fecal samples. The data showed significant differences in the gut microbial diversity of these three groups, and the α-diversity was the highest in wild males. Different factors influence the gut microbiota, and the main influencing factors were different in different groups, including sex differences, host genetics, and physical interactions. We also compared ecological assembly processes in the three groups. The results showed that mixed grouping contributed to the improvement of gut microbial diversity in domestic females. Our study provides effective and feasible suggestions for the feeding and management of the Datong yaks.

## 1. Introduction

Domesticated yaks (*Bos grunniens*) are an important source of meat, the main food for people on the Qinghai-Tibet Plateau [1]. The Datong yak is the first artificially cultivated breed of yaks globally, with good adaptability to high altitudes [2,3]. The Datong yak is the basis for genetic resource improvement of domestic yaks in the Qinghai-Tibet Plateau [3]. The health and reproductive status of Datong yaks are closely related to that of domesticated yaks on the Qinghai-Tibet Plateau. Datong yaks are mainly raised in the Datong yak breeding farm of Qinghai province in China, and the dominant method of rearing is mixed grouping, which means that all Datong yaks, including females, males, and wild males, are fed on the same pastures. The role of wild male yaks here is to improve or maintain the quality of calves.

The gut microbiota plays a pivotal role in the homeostasis and adaptation of yaks and can indicate the host’s health status [4,5,6,7]. Commonly, the main bacteria in the gut of yaks are Firmicutes and Bacteroidota, which are related to cellulose digestion, energy metabolism, and carbohydrate metabolism [8,9,10]. Proteobacteria, Actinobacteria, Verrucomicrobia, and Patescibacteria are also the main phyla in domestic yaks [11]. Different managements lead to differences in the diversity and function of the gut microbiota and affect the nutritional and health status of livestock [12,13]. Management practices can influence livestock health, causing variations in the gut microbiota. Understanding the characteristics of the gut microbiota of yaks under different managements can help improve the adaptive features and meat quality of yaks and is also an important monitoring measurement.

The fecal microbiota is a popular monitoring index requiring non-invasive sampling methods. In a mixed group, it is difficult to separate the main influencing factors, such as sex, distribution pattern, and environment [14,15,16,17,18,19,20].

Sex is the main factor influencing the diversity of the gut microbiota in males and females. However, its effects are often influenced by other factors [21,22,23]. Some studies show contrasting results [24,25]. The studies about Hainan typical wild boar and healthy Japanese people revealed significant differences in the diversity of the gut microbiota between males and females, which indicates that sex is a dominant factor that can influence the gut microbiota [23,24]. On the other hand, a study about Chinese forest musk deer showed no significant differences in the gut microbiota between males and females [25]. Several studies suggested that sexual dimorphism of the gut microbiota is associated with diseases, such as cardiovascular and cardiometabolic diseases [14,21,26,27]. Some other factors, including diet, age, sex hormones, and body mass index, influence the gut microbiota [28,29,30]. Sex differences are always overlooked [14].

The gut microbiota can be influenced by the surrounding environment, and hosts share the same gut microbiota when they live in the same area [16]. Sympatric chimpanzees and gorillas share 53% of the gut bacteria, and the gut microbiota can spread across species. If the geographical distance between species is short, a large amount of gut microorganisms can be exchanged [17,31]. The diversity and function of the gut microbiota differ from those of the microbiota in the environment [32,33]. Various studies observed different relationships between the gut microbiota and environment microorganisms [18,34]. The study of sympatric Amazonian wood-eating catfishes (Loricariidae) showed that the gut microbiota was determined by the host identity and phylogenetic history, but the gut microbiota of frog species from forest and farmland was influenced by the habitat [18,35].

The microbial ecological assembly process is a mechanism that shapes the diversity, function, and distribution of a microbial community. It is a main influencing factor of the gut microbiota [36]. The dominant process in the ecological assembly of the gut microbiota is stochastic and deterministic. The main factor of a deterministic process is abiotic, biotic, and environmental filtering, including interspecies interactions, pH, and temperature. The factors that shape microbial communities in stochastic processes include death, birth, and immigration, and microorganisms show a random balance between loss and gain [36,37,38]. Most studies on the microbial ecological assembly process focus on the environmental microbiota, while only a few studies focus on the gut microbiota [36,37,38,39].

The gut microbiota plays an important role in the health and immunity of animals. Monitoring the gut microbiota of Datong yaks by non-invasive fecal sampling can provide interesting information. As many factors influence the gut microbiota simultaneously, our study may help breeders and researchers identify the main factors.

In order to identify the main factors affecting the gut microbiota in a mixed group of yaks, in this study, fresh fecal samples were collected from Datong yaks and wild male yaks using a non-invasive method at the Datong yak breeding farm of Qinghai province in China. We compared the diversity and composition of the gut microbiota of female and male, wild and domesticated yaks which were housed in the same area based on the 16S rRNA V3–V4 regions. Our results lay the foundations for the management of domesticated yaks and provide constructive suggestions for the breeding of Datong yaks.

## 2. Materials and Methods

### 2.1. Sample Collection

All Datong yaks and wild male yaks were from the Datong yak breeding farm of Qinghai province (Datong, China) (37.25° N 101.37° E). A total of 26 fresh fecal samples were collected on 23 July 2021; of these, 10, 10, and 6 samples were obtained from domestic males, domestic females, and wild males, respectively. In the sampling area, all yaks grazed together. Because of the aggressive nature of wild males, they were kept in pens most of the time to avoid their escape. During sampling, we used disposable polyethylene (PE) gloves to avoid contamination, and one sample from one individual was placed in a Ziplock bag, numbered, and then stored at −80 °C in the Northwest Institution of Plateau Biology (Xining, Qinghai).

Yaks forage on pasture and are also provided with supplementary fodder in the Datong yak breeding farm of Qinghai province (Datong, China). Domestic males were fed a refined supplement feed specific for male yaks (Sanjiangyuan feed Factory, China), while domestic females were fed with a refined supplement feed specific for female yaks (Sanjiangyuan feed Factory, China), and wild males were fed another refined supplement feed specific for male yaks (Qinghai Hehuang feed Factory, China).

### 2.2. DNA Extraction, Amplification, and Sequencing

DNA library preparation and sequencing were performed at Majorbio Bio-Pharm Technology Co. Ltd., Shanghai, China. The E.Z.N.A.^®^ Soil DNA Kit (Omega Bio-tek, Norcross, GA, USA) was used for total DNA extraction following the manufacturer’s instructions. We performed 1% agarose gel electrophoresis to determine the extraction quality of the DNA. The quality of DNA was detected by a NanoDrop2000 UV–vis spectrophotometer (Thermo Scientific, Wilmington, NC, USA). The 16S rRNA V3–V4 regions were amplified with the primers 338F (5′-ACTCCTACGGGAGGCAGCAG-3′) and 806R (5′-GGACTACHVGGGTWTCTAAT-3′). The PCR conditions were as follows: 95 °C for 30 s, 95 °C for 3 min, 55 °C for 30 s for 27 cycles, 72 °C for 30 s, and 72 °C for 10 min. All PCR reactions were carried out in a 20 μL volume. PCR products from the same sample were mixed and purified using the AxyPrep DNA Gel Extraction Kit (Axygen Biosciences, Union City, CA, USA). Then, 2% agarose gel electrophoresis was used to assess all PCR products. The purified amplicons were sequenced on the Illumina’s Miseq PE300 platform (Illumina, San Diego, CA, USA). The raw data are available in the Sequence Read Archive (SRA) under the accession number PRJNA 825400 (https://www.ncbi.nlm.nih.gov/bioproject/PRJNA825400 (accessed on 11 April 2022)).

### 2.3. Data Processing

QIIME2-2020.2 was used for determining the bioinformatics pipeline. The obtained sequences were combined using FLASH (v1.2.11) software (http://ccb.jhu.edu/software/FLASH/, accessed on 11 April 2022), and sequence quality was filtered with fastp (0.19.6) (https://github.com/OpenGene/fastp), (accessed on 11 April 2022). after demultiplexing according to the particular barcode sequences of each sample [40,41]. To obtain the raw amplicon sequence variant (ASV) table and raw ASV representative sequences, DADA2 (through the q2-dada2 plugin) was used to denoise the sequences with default parameters [42].

The reference sequence annotation and curation pipeline (RESCRIPt) was used to construct a QIIME2-compatible amplicon-specific naive Bayes classifier to increase the quality of classification [43], based on the curated SILVA SSU NR99 (version 138) database (https://www.arb-silva.de/fileadmin/silva_databases/release_138/Exports/SILVA_138_SSURef_NR99_tax_silva.fasta.gz (accessed on 27 August 2021)), following a protocol suggested by the author (https://forum.qiime2.org/t/processing-filtering-and-evaluating-the-silva-database-and-other-reference-sequence-data-with-rescript/15494 (accessed on 27 August 2021)). The Q2-feature-classifier plugin was used to achieve taxonomic classification (0.8 confidence). The ASVs belonging to mitochondria, chloroplasts, or archaea were removed by taxonomic-based filtering. ASVs with a relative abundance of less than 0.01% and present in fewer than five samples were ruled out. The ASV table was normalized by the minimum sequencing depth of all samples for subsequent analysis.

In QIIME2 [44,45], per-sample ASV richness was computed using alpha diversity indices, covering Shannon and Simpson indices. The Venn diagram was analyzed using package ‘stats’ [46]. The Multcomp’ package in R and Rstudio was used to compute alpha (α)-diversity indices with the Kruskal–Wallis H test between different groups and the Tukey–Kramer post-hoc test [47]. The pairwise comparisons of the microbiota between any two groups at the phylum level, family level, and genus level were performed based on the Wilcoxon rank-sum test, using package ‘stats’ [46]. The q2-diversity-lib plugin in QIIME2 was used to perform all distance-based analyses based on the respective Bray–Curtis distances. The permutational multivariate analysis of variance (PERMANOVA) and the analysis of similarities (ANOSIM) based on Bray–Curtis distances were performed with the package ‘vegan’ and visualized with the package ‘ggplot2’ in R and Rstudio (Boston, MA, USA) [48,49]. All relevant analyses were performed using the free online Majorbio Cloud Platform (Shanghai Majorbio Bio-pharm Technology Co., Ltd., Shanghai, China).

### 2.4. Ecological Assembly Process of the Gut Microbiota

Using the modified stochasticity ratio, we calculated the contributions of stochastic and deterministic assembly processes to gut bacteria communities (MST). MST values higher than 0.5 and lower than 0.5 suggested that deterministic and stochastic assembly processes were prevalent [50]. The MST values were determined using the normalized stochasticity ratio (NST) package in R and Rstudio with 30,000 runs [50,51], following the protocol that the author suggested.

We looked at the departure of each observed metric from the null model’s average [checkerboard score (C-score)] to determine if the gut microbiotas were clustered or overdispersed [52]. The standardized effect size (SES) was determined using the null model, and standardized values were included for comparisons between assemblages. The C-score was calculated using the sequential swap randomization approach, using the ‘EcoSimR’ package in R and Rstudio (https://github.com/GotelliLab/EcoSimR/ (accessed on 13 April 2022), based on 30,000 simulations [37,52].

## 3. Results

### 3.1. Comparison of Gut Microbial Diversity among Domestic Females and Males and Wild Males

**Figure 1:**
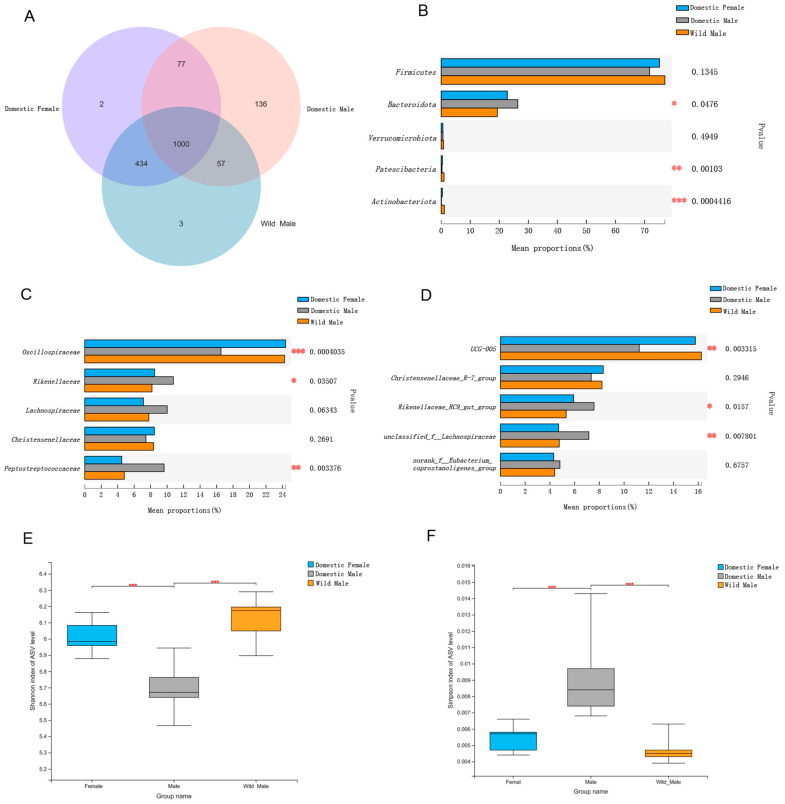
Comparison of gut microbial diversity among Datong yak domestic males and females and wild male yaks. (**A**) Venn diagram at amplicon sequence variant (ASV) level; (**B**) Top five phyla; (**C**) Top five families; (**D**) Top five genera; α-diversity in the gut microbiota; (**E**) Shannon indices at the ASV level; (**F**) Simpson indices at the ASV level. Significant differences in the gut microbiota in the three groups are indicated by *p* values and asterisks (* if 0.01 < *p* < 0.05, ** if 0.001 < *p* < 0.01, and *** if *p* < 0.001).

At the ASV level, a total of 1000 bacteria were shared among domestic females and males and wild males. The number of common bacteria between domestic females and wild males was the highest, with 434 ASVs. The number of ASVs specific to domestic males was the highest, with 136 ASVs (Figure 1A).

At the α-diversity level, the highest diversity of the gut microbiota was observed in wild males (Shannon = 6.12 ± 0.14; Simpson = 0.0047 ± 0.0008), followed by domestic females (Shannon = 6.01 ± 0.09; Simpson = 0.0054 ± 0.0007) and domestic males (Shannon = 5.70 ± 0.13; Simpson = 0.0089 ± 0.0023). Significant differences were observed in the gut microbial diversity among domestic females and males and wild males (*p* < 0.05). The gut microbial diversity in domestic and wild males and in domestic males and females showed significant differences (*p* < 0.05). However, there were no significant differences in gut microbial diversity between domestic females and wild males (*p* ≥ 0.05) (Figure 1E,F).

At the β-diversity level, significant differences were shown in gut microbial diversity among domestic females and males and wild males, based on ANOSIM analysis (R = 0.74, *p* = 0.001). PERMANOVA also showed the same results (Appendix A Table A1), indicating that inter-group differences were greater than intra-group differences (*p* = 0.001).

The comparisons at the β-diversity level showed that there were significant differences in gut microbial diversity between domestic males and wild males (R = 1, *p* = 0.001) and between domestic males and domestic females (R = 1, *p* = 0.001) based on ANOSIM analysis. There were no significant differences between domestic females and wild males (R = −0.05, *p* = 0.66) based on ANOSIM analysis. The results obtained by PERMANOVA were consistent with those of ANOSIM analysis (Appendix A Table A2, Table A3 and Table A4).

### 3.2. Comparison of Gut Microbial Composition among Domestic Females and Males and Wild Males

The main phyla in the gut microbiota of domestic females and males and in wild males were Firmicutes and Bacteroidota, whose combined relative abundance was more than 96%; there were no significant differences in Firmicutes in the above three groups (*p* > 0.05). The relative abundance of Firmicutes was greater than that of Bacteroidota. Among the top five phyla, only Bacteroidota, Patescibacteria, and Actinobacteriota showed significant differences in relative abundance (*p* < 0.05) (Figure 1B).

At the family level, the main family was Oscillospiraceae, whose relative abundance was more than 16% in all three groups. In addition to Oscillospiraceae, the relative abundances of Rikenellaceae, Lachnospiraceae, and Christensenellaceae were greater than 7%. Among the top five families, only Oscillospiraceae, Rikenellaceae, and Peptostreptococcaceae showed significant differences in the relative abundance in domestic females and males and wild males (*p* < 0.05) (Figure 1C).

At the genus level, the dominant genus was *UCG-005*, whose relative abundance was more than 11% in all three groups, and the lowest relative abundance was observed in the domestic males. *Christensenellaceae_R-7_group*, *Rikenellaceae_RC9_gut_group*, *unclassified_f__Lachnospiraceae*, and *norank_f__Eubacterium_coprostanoligenes_group* were the remaining the top five genera, with relative abundances above 7% in all three groups. Among the top five genera, only *UCG-005*, *Rikenellaceae_RC9_gut_group*, and *unclassified_f__Lachnospiraceae* showed significant differences in domestic females and males and wild males (*p* < 0.05) (Figure 1D).

### 3.3. Comparisons of Gut Microbial Composition in Domestic Females and Males and Wild Males

**Figure 2:**
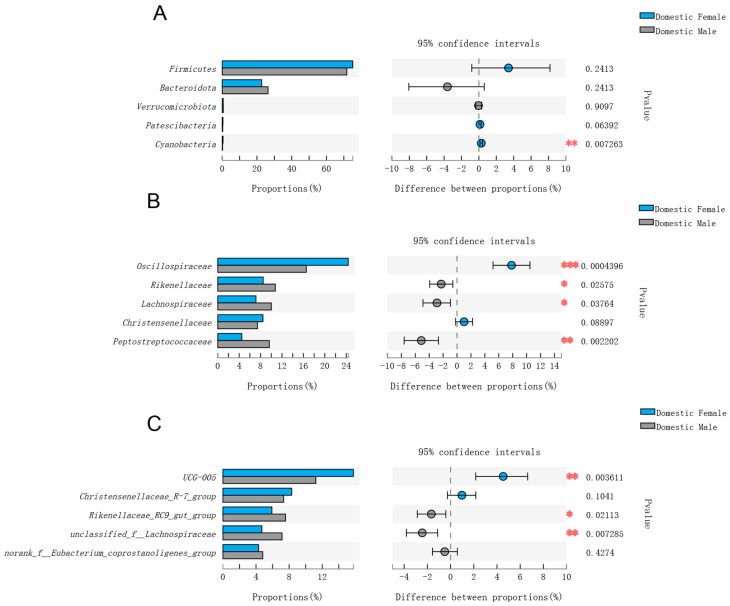
Comparison of gut microbial diversity between domestic females and males of Datong yaks. (**A**) Top five phyla; (**B**) top five families; (**C**) top five genera. Significant differences in the gut microbiota between domestic females and wild males of Datong yaks are indicated by *p* values and asterisks (* if 0.01 < *p* < 0.05, ** if 0.001 < *p* < 0.01, and *** if *p* < 0.001).

When comparing the relative abundances in the gut microbiota between domestic females and males, the relative abundances of Firmicutes, Patescibacteria, and Cyanobacteria were higher in domestic females, and the relative abundances of Bacteroidota and Verrucomicrobiota were lower in domestic females. In the top five phyla, only the relative abundance of Cyanobacteria showed a significant difference (*p* < 0.01) (Figure 2A). At the family level, the main family was Oscillospiraceae, whose relative abundance was significantly higher in domestic females than that in domestic males (*p* < 0.05). In the top five families, the relative abundances of Rikenellaceae, Lachnospiraceae, and Peptostreptococcaceae were significantly higher in domestic males (*p* < 0.05), and only the relative abundance of Christensenellaceae showed no significant difference in the three groups (*p* > 0.05) (Figure 2B). At the genus level, only the relative abundance of *UCG-005* was significantly higher in domestic females (*p* < 0.05). The relative abundances of *Rikenellaceae_RC9_gut_group* and *unclassified_f__Lachnospiraceae* were significantly higher in domestic males (*p* < 0.05) (Figure 2C).

**Figure 3:**
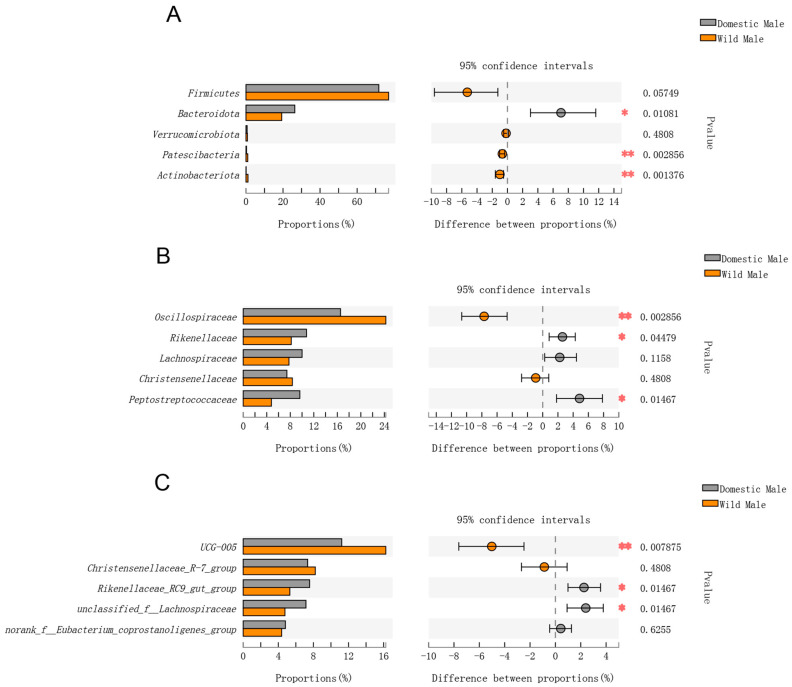
Comparison of gut microbial diversity between domestic Datong males and wild male yaks. (**A**) Top five phyla; (**B**) top five families; (**C**) top five genera. Significant differences in the gut microbiota between domestic Datong females and wild male yaks are indicated by *p* values and asterisks (* if 0.01 < *p* < 0.05, ** if 0.001 < *p* < 0.01).

When comparing the relative abundances of gut microorganisms between domestic males and wild males, only the relative abundance of Bacteroidota from the top five phyla was significantly higher in domestic males (*p* < 0.05). The relative abundances of Patescibacteria and Actinobacteriota were significantly higher in wild males (*p* < 0.05) (Figure 3A). At the family level, only the relative abundance of Oscillospiraceae was significantly higher in wild males (*p* < 0.01); this was the dominant family, whose relative abundance was more than 24% in wild males. The relative abundances of Rikenellaceae and Peptostreptococcaceae were significantly higher in domestic males (*p* < 0.05). The relative abundances of the rest of the top five families showed no significant differences (*p* > 0.05) (Figure 3B).

At the genus level, the relative abundance of the dominant genus, *UCG-005*, was significantly higher in wild males (*p* < 0.05). The relative abundances of *Rikenellaceae_RC9_gut_group* and *unclassified_f__Lachnospiraceae* were significantly higher in domestic males (*p* < 0.05) (Figure 3C).

**Figure 4:**
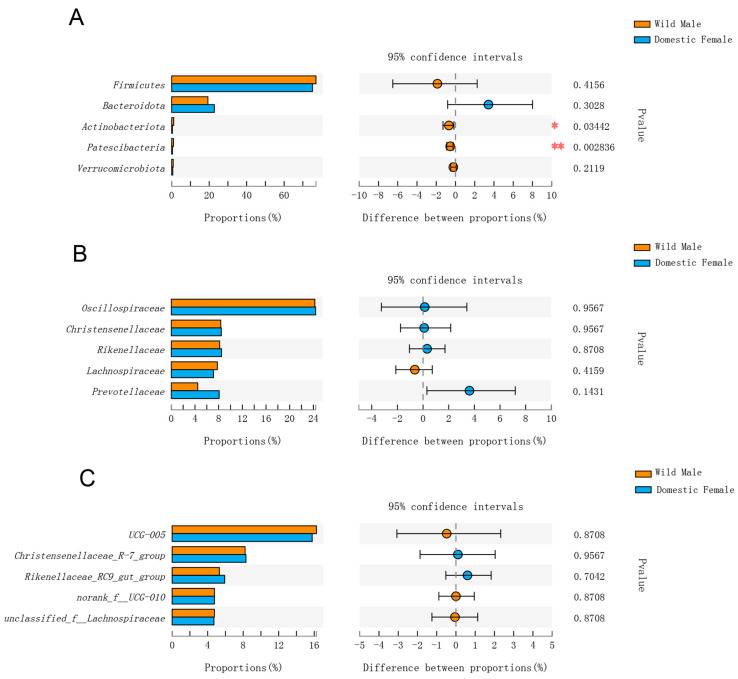
Comparison of gut microbial diversity between domestic Datong females and wild male yaks. (**A**) Top five phyla; (**B**) top five families; (**C**) top five genera. Significant differences in the gut microbiota between domestic Datong females and wild male yaks are indicated by *p* values and asterisks (* if 0.01 < *p* < 0.05, ** if 0.001 < *p* < 0.01).

When comparing the relative abundances of gut microorganisms between domestic females and wild males, the relative abundances of Actinobacteriota and Patescibacteria were significantly higher in wild males at the phylum level (*p* < 0.05), and the rest of the top five phyla showed no significant differences (*p* > 0.05) (Figure 4A). At the family level, only the relative abundance of Lachnospiraceae was higher in wild males. The top five families showed no significant differences between domestic females and wild males (*p* > 0.05) (Figure 4B). Of 69 comparative families, only 4 families showed significant differences between the two groups (*p* < 0.05), and their relative abundances were higher in wild males and less than 1%. At the genus level, there were no significant differences among the relative abundances of the top five genera (*p* > 0.05) (Figure 4C).

### 3.4. Ecological Assembly Process of the Gut Microbiota in Domestic Females and Males and in Wild Males

**Figure 5:**
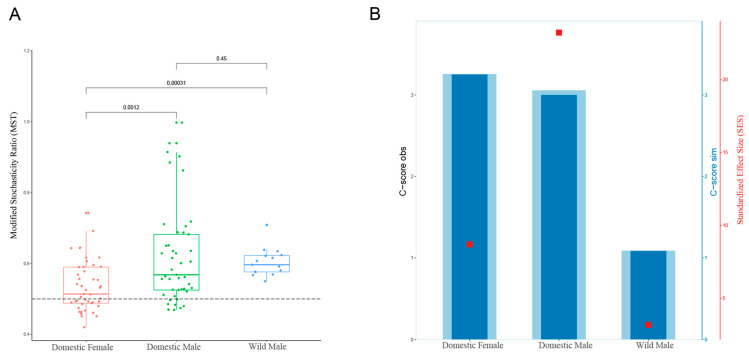
(**A**) Modified stochasticity ratio (MST) analysis and (**B**) checkerboard score (C-score) of the gut microbiota in domestic Datong male and female yaks and wild male yaks.

The values of MST in domestic females and males and in wild males were above 0.5, indicating that the dominant process was the stochastic process in all yaks. The values of SES revealed that the highest score was found for domestic males, which showed that the strongest influence of the deterministic process was observed in the gut microbiota of domestic males. The weakest influence was observed in the gut microbiota of wild males (Figure 5).

## 4. Discussion

### 4.1. Main Factors Influencing the Gut Microbial Diversity in Domestic Males and Females

There were significant differences in the gut microbial diversity in domestic Datong male and female yaks, which is consistent with a similar study in livestock [24]. Sex differences are the main factor influencing the gut microbiota, and the gut microbiota contributes to the sex differences. The metabolism of estrogen and androgen is related to gut microbial differences [30]. All samples collected from domestic males and females in this study were from adult yaks, and sex hormones shape gut microbial diversity [15]. Domestic male Datong yaks are not castrated, and androgen plays a significant role in the gut microbiota differences related to sex. There is mutual interaction between the gut microbiota and sex hormones [15,30,53].

The concentrations of four serum hormones in Datong yaks related to growth and metabolism, which are growth hormones (GHs), insulin, triiodothyronine, and thyroxine, at five 5 to 39 months of age, showed no significant differences between males and females [54]. As there are female and male Datong yaks in a mixed group, the domestic females in July will enter the estrus state when stimulated by the males [55]. The serum concentrations of reproductive hormones, including luteotropic hormone (LTH) and follicle-stimulating hormone (FSH), at estrus stages in female yaks were significantly higher than those at non-estrus stages [56,57]. Testosterone concentrations in males with offspring were higher during the rutting period than those during the non-reproductive season [58]. Therefore, the hormone levels of female and male yaks may differ during the estrus stage. The sex hormone levels are associated with gut microbiota diversity; thus, changes in sex hormone levels may lead to changes in gut microbial diversity [30].

### 4.2. Main Factors Influencing Gut Microbial Diversity in Domestic and Wild Males

Host genetics is the main factor influencing the gut microbial diversity, especially in different species [13,59]. Wild yaks are believed to be descended from the ancestors of domestic yaks, and the domestic yak is closely related to wild yaks. There is obvious genetic differentiation between domestic and wild yaks [60,61]. The domestic yak is the result of the domestication of wild yaks. For this reason, it is chosen by people. Gene composition and functional annotation results showed huge differences between domestic and wild yaks. There were differences mainly in reproduction, nutrition, and energy metabolism [61].

The distribution patterns of domestic and wild male yaks are also different. During the estrus stage, there is a competition for mating between wild and domestic males. Wild males have obvious mating advantages, as they are bigger and stronger than domestic males. Differences are also shown in the management system of domestic and wild males. Because wild males are more aggressive and wilder, they are sometimes kept in pens, while domestic males are usually free to range. Therefore, genetic differentiation and different distribution patterns are considered the main factors influencing the gut microbial diversity in domestic and wild males [31]. These two factors work together. Therefore, it is difficult to determine which one is more important.

### 4.3. Gut Microbial Diversity in Domestic Females and Wild Males during Estrus

There were no significant differences in gut microbial diversity between domestic females and wild males. We speculated that sex difference and host genetics are not the main factors influencing the gut microbiota. During estrus, domestic females and wild males interact more frequently, which means that this frequent interaction is the main reason for the similarity of their gut microbiotas [62,63]. A previous study reported that frequent physical interactions promoted and maintained similarities in the gut microbiota in red squirrels by maternal transmission [63]. A study about the gut microbial diversity of chimpanzees also supports these findings, indicating that frequent social interactions promote the spread of gut microbial diversity within and between host generations [64].

During estrus, domestic females like to have physical interactions with males [62]. As wild yaks have mating advantages, wild males have more physical interactions with domestic females than domestic males. Gut microbial diversity in domestic female yaks and wild male yaks showed no significant differences during estrus, but it is difficult to draw conclusions in the absence of other evidence. Based on our results, sex differences and host genetics cannot affect the gut microbial diversity in domestic female yaks and wild male yaks in a mixed group during estrus.

### 4.4. Effects of Different Gut Microbial Compositions on Domestic Females and Males and Wild Males

In the gut microbiota of domestic females and males and wild males, the dominant phyla were Firmicutes and Bacteroidetes. These two phyla are the common dominant bacteria of ungulates [34,65]. *Firmicutes* are mostly related to cellulose digestion to provide energy for the host, and *Bacteroidetes* mainly degrade carbohydrates and proteins to improve the utilization rates of these nutrients in the host [10,66]. The α-diversity of the gut microbiota in domestic females was significantly higher than that in domestic males, which is consistent with most studies [15,24]. Here, we speculated that sex is the dominant factor affecting the gut microbiota of domestic males and females. However, the α-diversity of the gut microbiota in wild males was significantly higher than that in domestic females, which is opposite to what is usually thought. Therefore, mixed grouping is considered the dominant factor shaping the gut microbiota, rather than sex differences between wild males and domestic females. Gut microbial diversity in wild and domestic animals showed significant differences. In general, the gut microbial diversity in domestic animals is lower than in wild animals [67]. A possible reason is domestication. Domestication influenced the gut microbial composition so that the gut microbial diversity is significantly lower in domestic yaks than in wild yaks [68].

### 4.5. Ecological Assembly Processes Reflecting the Living Status of Datong Yaks

Stochastic processes were the dominant processes influencing domestic females and males and wild males. The results of the C-score showed that the influence of stochastic processes was the strongest in wild males and the weakest in domestic males. In the gut microbiota of domestic males, intra-species competitions are highly observed. In the gut microbiota of wild males, the influences of all factors, including random births and deaths, are equal [37,39]. Homeostasis in wild males may be better maintained than in domestic males because it is less disturbed by external environmental factors, for example, temperature and pH, and the gut microbiota of wild males appeared in self-regulating equilibrium. The gut microbiota of domestic females appeared more influenced by wild males than domestic males. There may be bacteria exchange between domestic females and wild males. Thus, the homeostasis of domestic females was better maintained than that of domestic males. In addition, mixed grouping can improve the survival fitness of Datong yaks, and the ecological assembly processes of the gut microbiota may reflect the homeostasis of the host. However, there is limited information on ecological assembly processes of the gut microbiota. In the future, more studies are needed to explore these ecological assembly processes.

## 5. Conclusions

In this study, to determine the effects of mixed grouping on the gut microbiota of yaks, gut microbial diversity and ecological assembly processes were compared in domesticated male and female Datong yaks and wild male yaks living on the Qinghai-Tibet Plateau. In the condition of mixed grouping management, many factors work together affecting the gut microbiota of yaks, including sex differences, host genetics, and physical interactions. The dominant factors were different for different groups. The comparison of gut microbial diversity between domestic males and females showed that the dominant factor was hormones during estrus. The comparison between domestic males and wild males showed that main factors were both host genetics and distribution patterns. However, the comparison of the gut microbiota between domestic females and wild males showed that sex differences and host genetics were not the main factors; it may be social interactions played a dominant role. Although the effects of mixed grouping management on the gut microbiota of yaks are complicated, it is beneficial to improve the gut microbial diversity of domestic females. Since mixed groups are dominant, the determination of a cost-effective and efficient management method of Datong yaks on the Qinghai-Tibet Plateau and understanding the effects of mixed grouping on Datong yaks were our main aims. In this study, various factors, including sex, host genetic, and physical interactions, were analyzed. Diet is one of the main factors affecting gut microbial diversity. Although all Datong yaks were fed and foraged in the same area, their diets were very different. At present, there are no relevant studies on the differences between diets of Datong female and male yaks; therefore, the effects of differences in the diets of different groups on the gut microbiota should be studied in the future.

## Data Availability

The raw data are available on the Sequence Read Archive (SRA) under the accession number of Genbank PRJNA 825400 (https://www.ncbi.nlm.nih.gov/bioproject/PRJNA825400, accessed on 11 April 2022).

## Appendix A

**Table A1:** Results of the PERMANOVA analysis of the gut microbial diversities among domestic males, domestic females and wild males.

Name	Df	Sums of Sqs	MeanSqs	F.Models	R^2^	Pr(>F)
ALL Samples	2	1.7853	0.8927	8.5963	0.4278	0.001
Residuals	23	2.3884	0.1038	-	0.5722	-
Total	25	4.1737	-	-	1.0000	-

**Table A2:** Results of the PERMANOVA analysis of the gut microbial diversities between domestic males and females.

Name	Df	Sums of Sqs	MeanSqs	F.Models	R^2^	Pr(>F)
Domestic Males and Females	1	1.4024	1.4024	13.2632	0.4242	0.001
Residuals	18	1.9032	0.1057	-	0.5758	-
Total	19	3.3056	-	-	1.0000	-

**Table A3:** Results of the PERMANOVA analysis of the gut microbial diversities between domestic males and wild males.

Name	Df	Sums of Sqs	MeanSqs	F.Models	R^2^	Pr(>F)
Domestic Males and Wild Male	1	0.9339	0.9339	9.0800	0.4307	0.001
Residuals	12	1.2343	0.1029	-	0.5693	-
Total	13	2.1682	-	-	1.0000	-

**Table A4:** Results of the PERMANOVA analysis of the gut microbial diversities between domestic females and wild males.

Name	Df	Sums of Sqs	MeanSqs	F.Models	R^2^	Pr(>F)
Domestic Females and Wild Male	1	0.1145	0.1145	1.1479	0.0758	0.18
Residuals	14	1.3969	0.0998	-	0.9242	-
Total	15	1.5114	-	-	1.0000	-
